# Poly[[aqua­(μ_5_-3,4,5,6-tetra­carb­oxy­cyclo­hexane-1,2-dicarboxyl­ato)strontium] monohydrate]

**DOI:** 10.1107/S1600536811050811

**Published:** 2011-11-30

**Authors:** Pei-Chi Cheng, Jun-Xiang Zhan, Cheng-You Wu, Chia-Her Lin

**Affiliations:** a Department of Chemistry, R&D Center for Membrane Technology, Center for Nanotechnology, Chung-Yuan Christian University, Chung-Li 320, Taiwan; b Department of Chemistry, Chung-Yuan Christian University, Chung-Li 320, Taiwan

## Abstract

In the title compound, {[Sr(C_12_H_10_O_12_)(H_2_O)]·H_2_O}_*n*_, the Sr^II^ ion is coordinated by six O atoms of five symmetry-related 3,4,5,6-tetra­carb­oxy­cyclo­hexane-1,2-dicarboxyl­ate ligands and one water mol­ecule in a slightly distorted monocapped trigonal–prismatic environment. The ligands bridge the Sr^II^ ions, forming a two-dimensional structure. In the crystal, O—H⋯O hydrogen bonds further connect the structure into a three-dimensional network. The H atoms of two of the carboxyl groups were refined as half-occupancy.

## Related literature

For general background to coordination polymers, see: Liu *et al.* (2009 ▶); Liang *et al.* (2011 ▶); Kitagawa *et al.* (2004 ▶); Jiang & Xu (2011 ▶). For details of compounds based on cyclo­hexane-1,2,3,4,5,6-hexacarboxylic acid, see: Canadillas-Delgado *et al.* (2010 ▶). For related structures, see: Che *et al.* (2006 ▶); Yu *et al.* (2007 ▶); Chen & Meng (2010 ▶).
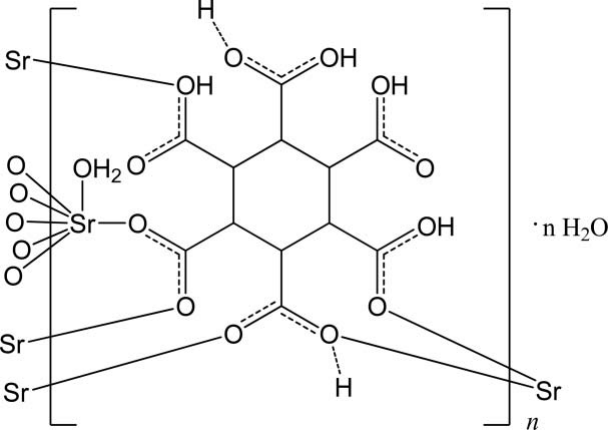


## Experimental

### 

#### Crystal data

[Sr(C_12_H_10_O_12_)(H_2_O)]·H_2_O
*M* *_r_* = 469.85
Triclinic, 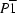

*a* = 6.1583 (3) Å
*b* = 9.4491 (3) Å
*c* = 13.6710 (5) Å
α = 77.614 (2)°
β = 80.746 (2)°
γ = 77.041 (2)°
*V* = 751.89 (5) Å^3^
*Z* = 2
Mo *K*α radiation
μ = 3.67 mm^−1^
*T* = 296 K
0.15 × 0.12 × 0.10 mm

#### Data collection

Bruker APEXII CCD diffractometer
Absorption correction: multi-scan (*SADABS*; Bruker, 2010 ▶) *T*_min_ = 0.609, *T*_max_ = 0.710
8798 measured reflections
3616 independent reflections
3184 reflections with *I* > 2σ(*I*)
*R*_int_ = 0.027

#### Refinement

*R*[*F* ^2^ > 2σ(*F* ^2^)] = 0.028
*wR*(*F* ^2^) = 0.065
*S* = 1.04
3616 reflections
244 parameters
H-atom parameters constrained
Δρ_max_ = 0.45 e Å^−3^
Δρ_min_ = −0.55 e Å^−3^

### 

Data collection: *APEX2* (Bruker, 2010 ▶); cell refinement: *SAINT* (Bruker, 2010 ▶); data reduction: *SAINT*; program(s) used to solve structure: *SHELXS97* (Sheldrick, 2008 ▶); program(s) used to refine structure: *SHELXL97* (Sheldrick, 2008 ▶); molecular graphics: *DIAMOND* (Brandenburg, 2010 ▶); software used to prepare material for publication: *SHELXTL* (Sheldrick, 2008 ▶).

## Supplementary Material

Crystal structure: contains datablock(s) I, global. DOI: 10.1107/S1600536811050811/lh5373sup1.cif

Structure factors: contains datablock(s) I. DOI: 10.1107/S1600536811050811/lh5373Isup2.hkl

Additional supplementary materials:  crystallographic information; 3D view; checkCIF report

## Figures and Tables

**Table 1:** Hydrogen-bond geometry (Å, °)

*D*—H⋯*A*	*D*—H	H⋯*A*	*D*⋯*A*	*D*—H⋯*A*
O4—H4*A*⋯O4^i^	0.82	1.68	2.493 (3)	172
O5—H11⋯O1	0.82	1.79	2.596 (2)	167
O8—H7⋯O12	0.82	1.75	2.549 (2)	163
O9—H9*A*⋯O9^ii^	0.82	1.65	2.457 (3)	168.6
O10—H9⋯O1*W*^iii^	0.82	1.72	2.533 (2)	174
O13—H13*A*⋯O11^iv^	0.85	1.97	2.820 (2)	175
O13—H13*B*⋯O2^v^	0.85	2.22	3.035 (3)	160
O1*W*—H1*WB*⋯O7^vi^	0.85	2.03	2.836 (3)	159
O1*W*—H1*WB*⋯O7^vi^	0.85	2.03	2.836 (3)	159

Symmetry codes: (i) 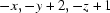; (ii) 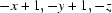; (iii) 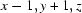; (iv) 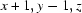; (v) 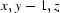; (vi) 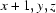.

**Fig. 1.**
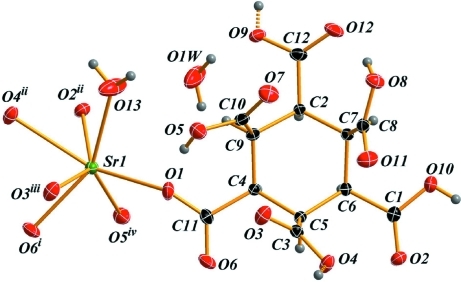
A view of the title compound, showing 50% probability displacement ellipsoids. [symmetry codes: (i) -x + 2, -y + 1, -z + 1;(ii) x + 1, y - 1, z; (iii) -x + 1, -y + 1, -z + 1; (iv) x + 1, y, z].

**Fig. 2.**
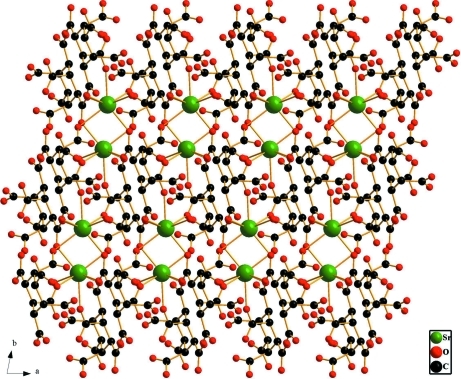
The layer structure of the title compound viewed along the b axis.

## supplementary crystallographic information

### Comment

The synthesis of coordination polymers *via* multidentate ligands have received considerable attention, owing to their novel structures and special functional properties (Liu *et al.*, 2009; Liang *et al.*, 2011; Kitagawa *et al.*, 2004; Jiang & Xu, 2011). cyclohexane-1,2,3,4,5,6-hexacarboxylate acid is a flexible ligand for constructing new coordination compounds (Canadillas-Delgado *et al.*, 2010). The structure of the title complex formed from the reaction of this acid with Sr^II^ ion is reported herein. Some related structures have already appeared in the literature (Che *et al.*, 2006; Yu *et al.* 2007; Chen *et al.*, 2010).

The asymmetric unit of the title compound is shown in Fig. 1. The Sr^II^ ion atom is seven-coordinated by six oxygen atoms of five carboxylate ligands and one oxygen atom of coordinated water molecules. The Sr—O distances from 2.4614 (19) to 2.7043 (17) Å. The Sr^II^ ions are connected *via* the ligands into a extended two-dimensional layer (Fig. 2). There are hydrogen bonding interactions involving the water molecules and some carboxyl O atoms.

### Experimental

Solvothermal reactions were carried out at 363 K for 2 d in a Teflon-lined acid digestion bomb with an internal volume of 23 ml followed by slow cooling at 6 K/h to room temperature. A single-phase product consisting of colorless crystals of was obtained from a mixture of cyclohexane-1,2,3,4,5,6-hexacarboxylate acid (C_12_H_12_O_12_, 0.0348 g, 0.1 mmol), Sr(NO_3_)_2_ (0.0635 g, 0.3 mmol), and ethanol (5.0 ml) and H_2_O (1.0 ml).

### Refinement

H atoms were constrained to ideal geometries, with C—H = 0.98 Å, O—H = 0.82–0.85 Å and *U*_iso_(H) = 1.2*U*_eq_(C);*U*_iso_(H) = 1.5*U*_eq_(O). The the H atoms of the carboxylic acid groups groups containing O4 and O9 were refined as half occupancy. This is determined by the inversion symmetry realtionship which which cause unrealistic short H···H distances for full occupancy H atoms. The carboxylic acid H atom positions were included on the basis of sensible hydrogen bonds, the longer C-O distance in the group and the non-coordination to Sr.

### Crystal data

**Table:**

[Sr(C_12_H_10_O_12_)(H_2_O)]·H_2_O	*Z* = 2
*M**_r_* = 469.85	*F*(000) = 472
Triclinic, *P*1	*D*_x_ = 2.075 Mg m^−^^3^
Hall symbol: -P 1	Mo *K*α radiation, λ = 0.71073 Å
*a* = 6.1583 (3) Å	Cell parameters from 5438 reflections
*b* = 9.4491 (3) Å	θ = 2.5–28.4°
*c* = 13.6710 (5) Å	µ = 3.67 mm^−^^1^
α = 77.614 (2)°	*T* = 296 K
β = 80.746 (2)°	Columnar, colourless
γ = 77.041 (2)°	0.15 × 0.12 × 0.10 mm
*V* = 751.89 (5) Å^3^	

### Data collection

**Table:**

Bruker APEXII CCD diffractometer	3616 independent reflections
Radiation source: fine-focus sealed tube	3184 reflections with *I* > 2σ(*I*)
graphite	*R*_int_ = 0.027
Detector resolution: 8.3333 pixels mm^-1^	θ_max_ = 28.4°, θ_min_ = 1.5°
φ and ω scans	*h* = −7→7
Absorption correction: multi-scan (*SADABS*; Bruker, 2010)	*k* = −12→12
*T*_min_ = 0.609, *T*_max_ = 0.710	*l* = −18→18
8798 measured reflections	

### Refinement

**Table:**

Refinement on *F*^2^	Primary atom site location: structure-invariant direct methods
Least-squares matrix: full	Secondary atom site location: difference Fourier map
*R*[*F*^2^ > 2σ(*F*^2^)] = 0.028	Hydrogen site location: inferred from neighbouring sites
*wR*(*F*^2^) = 0.065	H-atom parameters constrained
*S* = 1.04	*w* = 1/[σ^2^(*F*_o_^2^) + (0.0333*P*)^2^ + 0.1009*P*] where *P* = (*F*_o_^2^ + 2*F*_c_^2^)/3
3616 reflections	(Δ/σ)_max_ = 0.001
244 parameters	Δρ_max_ = 0.45 e Å^−^^3^
0 restraints	Δρ_min_ = −0.55 e Å^−^^3^

### Special details

**Table:**

Geometry. All e.s.d.'s (except the e.s.d. in the dihedral angle between two l.s. planes) are estimated using the full covariance matrix. The cell e.s.d.'s are taken into account individually in the estimation of e.s.d.'s in distances, angles and torsion angles; correlations between e.s.d.'s in cell parameters are only used when they are defined by crystal symmetry. An approximate (isotropic) treatment of cell e.s.d.'s is used for estimating e.s.d.'s involving l.s. planes.
Refinement. Refinement of *F*^2^ against ALL reflections. The weighted *R*-factor *wR* and goodness of fit *S* are based on *F*^2^, conventional *R*-factors *R* are based on *F*, with *F* set to zero for negative *F*^2^. The threshold expression of *F*^2^ > σ(*F*^2^) is used only for calculating *R*-factors(gt) *etc*. and is not relevant to the choice of reflections for refinement. *R*-factors based on *F*^2^ are statistically about twice as large as those based on *F*, and *R*- factors based on ALL data will be even larger.

### Fractional atomic coordinates and isotropic or equivalent isotropic displacement parameters (Å^2^)

**Table:**

	*x*	*y*	*z*	*U*_iso_*/*U*_eq_	Occ. (<1)
Sr1	1.02988 (3)	0.31958 (2)	0.409253 (15)	0.01534 (7)	
O1	0.7024 (3)	0.53526 (16)	0.41114 (12)	0.0235 (4)	
O2	0.3026 (3)	1.24350 (16)	0.26117 (12)	0.0216 (4)	
O3	0.1971 (3)	0.78708 (16)	0.43110 (12)	0.0226 (4)	
O4	0.1356 (3)	1.02924 (16)	0.42401 (12)	0.0221 (4)	
H4A	0.0559	1.0101	0.4772	0.033*	0.50
O5	0.3286 (3)	0.50144 (16)	0.36308 (12)	0.0246 (4)	
H11	0.4481	0.4983	0.3833	0.037*	
O6	0.7105 (3)	0.74239 (18)	0.46176 (13)	0.0294 (4)	
O7	0.1292 (3)	0.63238 (18)	0.24436 (13)	0.0276 (4)	
O8	−0.0422 (3)	0.90185 (17)	0.09955 (12)	0.0233 (4)	
H7	0.0502	0.8645	0.0574	0.035*	
O9	0.5394 (3)	0.56570 (17)	0.06154 (13)	0.0264 (4)	
H9A	0.5248	0.5275	0.0151	0.025*	0.50
O10	0.1959 (3)	1.22482 (17)	0.11758 (13)	0.0273 (4)	
H9	0.1282	1.3095	0.1200	0.041*	
O11	−0.0412 (3)	0.98476 (18)	0.23773 (13)	0.0250 (4)	
O12	0.2765 (3)	0.7502 (2)	−0.00060 (14)	0.0360 (5)	
O13	0.7610 (3)	0.2429 (2)	0.31616 (18)	0.0494 (6)	
H13A	0.8148	0.1626	0.2948	0.074*	
H13B	0.6253	0.2656	0.3039	0.074*	
C1	0.2938 (4)	1.1700 (2)	0.19893 (16)	0.0150 (4)	
C2	0.4656 (4)	0.7781 (2)	0.13407 (15)	0.0130 (4)	
H2	0.6144	0.7997	0.1072	0.016*	
C3	0.2404 (4)	0.9094 (2)	0.39319 (15)	0.0142 (4)	
C4	0.5861 (4)	0.7676 (2)	0.30273 (15)	0.0125 (4)	
H5	0.7244	0.7907	0.2621	0.015*	
C5	0.4433 (3)	0.9213 (2)	0.31267 (15)	0.0121 (4)	
H1	0.5384	0.9716	0.3387	0.015*	
C6	0.4077 (3)	1.0093 (2)	0.20485 (15)	0.0125 (4)	
H6	0.5604	1.0140	0.1722	0.015*	
C7	0.3150 (4)	0.9317 (2)	0.13770 (15)	0.0132 (4)	
H4	0.3348	0.9892	0.0693	0.016*	
C8	0.0628 (4)	0.9391 (2)	0.16407 (16)	0.0164 (4)	
C9	0.4994 (4)	0.6810 (2)	0.23850 (15)	0.0137 (4)	
H3	0.6245	0.5998	0.2260	0.016*	
C10	0.3007 (4)	0.6079 (2)	0.28292 (16)	0.0173 (5)	
C11	0.6685 (4)	0.6760 (2)	0.40099 (16)	0.0162 (4)	
C12	0.4151 (4)	0.6951 (2)	0.05943 (17)	0.0198 (5)	
O1W	0.9816 (4)	0.4880 (2)	0.11301 (19)	0.0547 (6)	
H1WA	0.8583	0.4974	0.0897	0.082*	
H1WB	1.0208	0.5503	0.1398	0.082*	

### Atomic displacement parameters (Å^2^)

**Table:**

	*U*^11^	*U*^22^	*U*^33^	*U*^12^	*U*^13^	*U*^23^
Sr1	0.01520 (12)	0.01490 (10)	0.01647 (11)	−0.00211 (7)	−0.00322 (7)	−0.00400 (7)
O1	0.0279 (10)	0.0149 (7)	0.0235 (9)	0.0039 (6)	−0.0068 (7)	−0.0004 (6)
O2	0.0268 (10)	0.0165 (7)	0.0224 (8)	−0.0022 (6)	−0.0029 (7)	−0.0078 (6)
O3	0.0235 (9)	0.0176 (7)	0.0239 (8)	−0.0062 (7)	0.0073 (7)	−0.0030 (6)
O4	0.0257 (10)	0.0160 (7)	0.0210 (8)	−0.0014 (6)	0.0082 (7)	−0.0066 (6)
O5	0.0317 (10)	0.0218 (8)	0.0235 (9)	−0.0150 (7)	−0.0070 (7)	0.0020 (6)
O6	0.0419 (12)	0.0280 (9)	0.0238 (9)	−0.0112 (8)	−0.0203 (8)	−0.0004 (7)
O7	0.0199 (10)	0.0337 (9)	0.0324 (10)	−0.0093 (7)	−0.0059 (8)	−0.0067 (7)
O8	0.0179 (9)	0.0312 (9)	0.0231 (9)	−0.0029 (7)	−0.0061 (7)	−0.0090 (7)
O9	0.0321 (11)	0.0214 (8)	0.0297 (9)	0.0048 (7)	−0.0133 (7)	−0.0164 (7)
O10	0.0382 (11)	0.0152 (7)	0.0274 (9)	0.0043 (7)	−0.0144 (8)	−0.0039 (7)
O11	0.0172 (9)	0.0312 (9)	0.0285 (9)	−0.0048 (7)	0.0031 (7)	−0.0138 (7)
O12	0.0388 (12)	0.0400 (10)	0.0320 (10)	0.0139 (9)	−0.0240 (9)	−0.0210 (8)
O13	0.0267 (12)	0.0530 (13)	0.0824 (17)	0.0043 (9)	−0.0197 (11)	−0.0454 (12)
C1	0.0118 (11)	0.0153 (9)	0.0168 (10)	−0.0035 (8)	0.0013 (8)	−0.0018 (8)
C2	0.0145 (11)	0.0127 (9)	0.0115 (9)	−0.0004 (8)	−0.0019 (8)	−0.0035 (7)
C3	0.0125 (11)	0.0169 (9)	0.0131 (10)	−0.0012 (8)	−0.0024 (8)	−0.0035 (8)
C4	0.0124 (11)	0.0117 (9)	0.0137 (10)	−0.0016 (8)	−0.0025 (8)	−0.0030 (7)
C5	0.0103 (11)	0.0122 (9)	0.0146 (10)	−0.0022 (7)	−0.0020 (8)	−0.0037 (7)
C6	0.0102 (11)	0.0134 (9)	0.0130 (10)	−0.0015 (8)	−0.0005 (8)	−0.0020 (7)
C7	0.0151 (12)	0.0132 (9)	0.0116 (9)	−0.0030 (8)	−0.0017 (8)	−0.0025 (7)
C8	0.0166 (12)	0.0137 (9)	0.0178 (11)	−0.0010 (8)	−0.0041 (9)	−0.0012 (8)
C9	0.0133 (11)	0.0141 (9)	0.0136 (10)	−0.0018 (8)	0.0000 (8)	−0.0046 (7)
C10	0.0228 (13)	0.0142 (9)	0.0169 (11)	−0.0058 (9)	−0.0012 (9)	−0.0056 (8)
C11	0.0115 (11)	0.0194 (10)	0.0167 (10)	−0.0016 (8)	−0.0036 (8)	−0.0012 (8)
C12	0.0210 (13)	0.0241 (11)	0.0161 (11)	−0.0028 (9)	−0.0019 (9)	−0.0098 (9)
O1W	0.0434 (14)	0.0313 (11)	0.0981 (19)	0.0114 (9)	−0.0392 (13)	−0.0280 (11)

### Geometric parameters (Å, °)

**Table:**

Sr1—O6^i^	2.4601 (17)	O10—H9	0.8200
Sr1—O1	2.5267 (15)	O11—C8	1.210 (3)
Sr1—O2^ii^	2.5348 (15)	O12—C12	1.231 (3)
Sr1—O3^iii^	2.5378 (14)	O13—H13A	0.8499
Sr1—O13	2.549 (2)	O13—H13B	0.8497
Sr1—O4^ii^	2.6448 (14)	C1—C6	1.513 (3)
Sr1—O5^iv^	2.7043 (15)	C2—C12	1.519 (3)
O1—C11	1.280 (2)	C2—C9	1.540 (3)
O2—C1	1.223 (3)	C2—C7	1.543 (3)
O2—Sr1^v^	2.5348 (15)	C2—H2	0.9800
O3—C3	1.229 (2)	C3—C5	1.534 (3)
O3—Sr1^iii^	2.5378 (14)	C4—C11	1.528 (3)
O4—C3	1.291 (2)	C4—C5	1.541 (3)
O4—Sr1^v^	2.6448 (14)	C4—C9	1.543 (3)
O4—H4A	0.8194	C4—H5	0.9800
O5—C10	1.324 (3)	C5—C6	1.552 (3)
O5—Sr1^vi^	2.7043 (15)	C5—H1	0.9800
O5—H11	0.8197	C6—C7	1.534 (3)
O6—C11	1.231 (3)	C6—H6	0.9800
O6—Sr1^i^	2.4600 (17)	C7—C8	1.526 (3)
O7—C10	1.210 (3)	C7—H4	0.9800
O8—C8	1.319 (3)	C9—C10	1.518 (3)
O8—H7	0.8199	C9—H3	0.9800
O9—C12	1.286 (3)	O1W—H1WA	0.8494
O9—H9A	0.8204	O1W—H1WB	0.8507
O10—C1	1.303 (3)		
			
O6^i^—Sr1—O1	120.71 (6)	C12—C2—H2	104.0
O6^i^—Sr1—O2^ii^	99.27 (6)	C9—C2—H2	104.0
O1—Sr1—O2^ii^	129.08 (5)	C7—C2—H2	104.0
O6^i^—Sr1—O3^iii^	75.76 (6)	O3—C3—O4	123.21 (19)
O1—Sr1—O3^iii^	82.17 (5)	O3—C3—C5	119.48 (18)
O2^ii^—Sr1—O3^iii^	141.73 (5)	O4—C3—C5	117.00 (17)
O6^i^—Sr1—O13	149.80 (7)	C11—C4—C5	114.33 (16)
O1—Sr1—O13	78.85 (6)	C11—C4—C9	114.86 (16)
O2^ii^—Sr1—O13	80.94 (6)	C5—C4—C9	115.82 (18)
O3^iii^—Sr1—O13	85.48 (7)	C11—C4—H5	103.1
O6^i^—Sr1—O4^ii^	80.20 (5)	C5—C4—H5	103.1
O1—Sr1—O4^ii^	143.19 (5)	C9—C4—H5	103.1
O2^ii^—Sr1—O4^ii^	67.90 (5)	C3—C5—C4	111.23 (15)
O3^iii^—Sr1—O4^ii^	73.88 (5)	C3—C5—C6	119.73 (18)
O13—Sr1—O4^ii^	71.87 (6)	C4—C5—C6	107.85 (15)
O6^i^—Sr1—O5^iv^	69.92 (5)	C3—C5—H1	105.7
O1—Sr1—O5^iv^	91.80 (5)	C4—C5—H1	105.7
O2^ii^—Sr1—O5^iv^	72.48 (5)	C6—C5—H1	105.7
O3^iii^—Sr1—O5^iv^	135.64 (5)	C1—C6—C7	112.61 (18)
O13—Sr1—O5^iv^	136.54 (7)	C1—C6—C5	115.32 (16)
O4^ii^—Sr1—O5^iv^	124.84 (5)	C7—C6—C5	115.75 (16)
O6^i^—Sr1—Sr1^i^	65.88 (4)	C1—C6—H6	103.7
O1—Sr1—Sr1^i^	56.51 (4)	C7—C6—H6	103.7
O2^ii^—Sr1—Sr1^i^	132.72 (4)	C5—C6—H6	103.7
O3^iii^—Sr1—Sr1^i^	80.63 (3)	C8—C7—C6	111.99 (16)
O13—Sr1—Sr1^i^	134.51 (4)	C8—C7—C2	117.48 (16)
O4^ii^—Sr1—Sr1^i^	141.65 (4)	C6—C7—C2	109.12 (18)
O5^iv^—Sr1—Sr1^i^	60.24 (3)	C8—C7—H4	105.8
C11—O1—Sr1	138.52 (15)	C6—C7—H4	105.8
C1—O2—Sr1^v^	132.64 (14)	C2—C7—H4	105.8
C3—O3—Sr1^iii^	135.56 (13)	O11—C8—O8	120.5 (2)
C3—O4—Sr1^v^	153.52 (13)	O11—C8—C7	122.3 (2)
C3—O4—H4A	110.5	O8—C8—C7	117.08 (18)
Sr1^v^—O4—H4A	95.9	C10—C9—C2	111.94 (19)
C10—O5—Sr1^vi^	116.67 (14)	C10—C9—C4	119.05 (16)
C10—O5—H11	109.5	C2—C9—C4	108.95 (16)
Sr1^vi^—O5—H11	133.1	C10—C9—H3	105.2
C11—O6—Sr1^i^	134.49 (15)	C2—C9—H3	105.2
C8—O8—H7	109.5	C4—C9—H3	105.2
C12—O9—H9A	112.4	O7—C10—O5	119.0 (2)
C1—O10—H9	109.5	O7—C10—C9	123.8 (2)
Sr1—O13—H13A	113.5	O5—C10—C9	116.8 (2)
Sr1—O13—H13B	140.9	O6—C11—O1	124.7 (2)
H13A—O13—H13B	105.1	O6—C11—C4	117.76 (18)
O2—C1—O10	123.08 (19)	O1—C11—C4	117.38 (18)
O2—C1—C6	123.6 (2)	O12—C12—O9	123.6 (2)
O10—C1—C6	113.16 (18)	O12—C12—C2	122.80 (19)
C12—C2—C9	113.13 (16)	O9—C12—C2	113.5 (2)
C12—C2—C7	115.66 (19)	H1WA—O1W—H1WB	126.9
C9—C2—C7	114.16 (16)		

Symmetry codes: (i) −*x*+2, −*y*+1, −*z*+1; (ii) *x*+1, *y*−1, *z*; (iii) −*x*+1, −*y*+1, −*z*+1; (iv) *x*+1, *y*, *z*; (v) *x*−1, *y*+1, *z*; (vi) *x*−1, *y*, *z*.

### Hydrogen-bond geometry (Å, °)

**Table:**

*D*—H···*A*	*D*—H	H···*A*	*D*···*A*	*D*—H···*A*
O4—H4A···O4^vii^	0.82	1.68	2.493 (3)	172.
O5—H11···O1	0.82	1.79	2.596 (2)	167.
O8—H7···O12	0.82	1.75	2.549 (2)	163.
O9—H9A···O9^viii^	0.82	1.65	2.457 (3)	168.6
O10—H9···O1W^v^	0.82	1.72	2.533 (2)	174.
O13—H13A···O11^ii^	0.85	1.97	2.820 (2)	175.
O13—H13B···O2^ix^	0.85	2.22	3.035 (3)	160.
O1W—H1WB···O7^iv^	0.85	2.03	2.836 (3)	159.
O1W—H1WB···O7^iv^	0.85	2.03	2.836 (3)	159.

Symmetry codes: (vii) −*x*, −*y*+2, −*z*+1; (viii) −*x*+1, −*y*+1, −*z*; (v) *x*−1, *y*+1, *z*; (ii) *x*+1, *y*−1, *z*; (ix) *x*, *y*−1, *z*; (iv) *x*+1, *y*, *z*.
